# Polyurea for Blast and Impact Protection: A Review

**DOI:** 10.3390/polym14132670

**Published:** 2022-06-30

**Authors:** Rui Zhang, Weibo Huang, Ping Lyu, Shuai Yan, Xu Wang, Jiahui Ju

**Affiliations:** 1 School of Civil Engineering, Qingdao University of Technology, Qingdao 266520, China; zhangray126@126.com (R.Z.); yanshuai1506@163.com (S.Y.); wangxu7056@163.com (X.W.); 2 Qingdao Shamu Advanced Material Co., Ltd., Qingdao 266000, China; jujiahui2015@163.com

**Keywords:** blast protection, impact, polyurea, reinforcement

## Abstract

Polyurea has attracted extensive attention from researchers and engineers in the field of blast and impact protection due to its excellent quasi-static mechanical properties and dynamic mechanical properties. Its mechanical properties and energy absorption capacity have been tuned by means of formulation optimization, molecular dynamics (MD) simulation and the addition of reinforcing materials. Owing to the special molecular structure of polyurea, the mechanism of polyurea protection against blasts and impacts is the simultaneous effect of multiple properties. For different substrates and structures, polyurea needs to provide different performance characteristics, including adhesion, hardness, breaking elongation, etc., depending on the characteristics of the load to which it is subjected. The current article reviews relevant publications in the field of polyurea blast and impact protection, including material optimization, protection mechanisms and applications in blast and impact protection.

## 1. Introduction

**Scheme 1:**
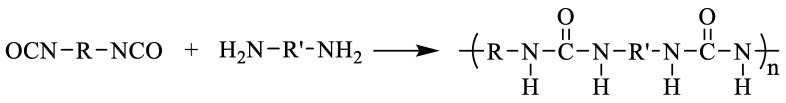
The reaction of isocyanate prepolymer with polyamine.

Polyurea is a block copolymer synthesized from the rapid reaction between isocyanate prepolymers and polyamines [1,2,3,4,5,6,7], as shown in Scheme 1. The commercial polyurea formulation consists of two components, A and B, where the isocyanate prepolymer is component A, and component B consists of a homogeneous mixture of long polyether amines, chain extenders and additives.

**Figure 1:**
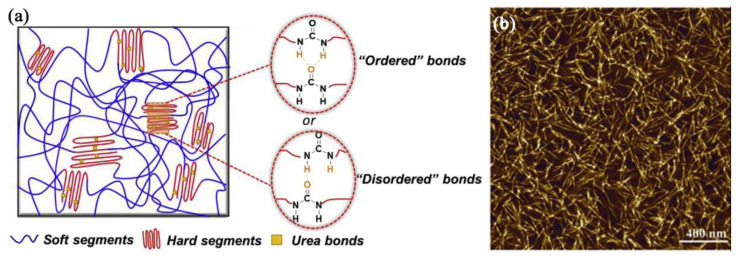
Structure of segmented polyurea: (**a**) Simplified structure; (**b**) AFM tapping mode phase images of polyurea. Reproduced with permission from ref. [2].

A unique microphase separation can be observed in the polymer microstructure due to the existence of soft and hard segments [2,8,9,10], as shown in Figure 1a. The microstructure shows that the hard segments of the crosslinked mesh structure are uniformly distributed among the soft segments of the matrix, as shown in Figure 1b, where the bright regions are the hard segments. Given this microphase-separated structure, polyurea can be considered a nano-composite, with the hard segments acting as reinforcements dispersed in a soft-segment matrix [11]. Such a special microstructure gives rise to excellent macroscopic properties, such as stability, high strength and aging resistance.

Spray polyurea elastomer technology was developed by Primeaux in the 1980s as a rapid prototyping polyurea system based on reaction injection molding (RIM), successfully applied to the preparation of both aromatic and aliphatic spray polyurea elastomers [7,12]. It is known as a versatile coating technology and is rapidly being commercially promoted [7]. Polyurea protects different substrates against moisture, micro-organisms and weeds, extending the life of the structure. With a high energy-absorbing capacity, polyurea is also used as a damping and protective material for ramps, driveways and cargo spaces. High chemical resistance to bases, organic acids up to a concentration of 10%, inorganic acids up to a concentration of 20% and their salts make them applicable for the protection of tanks used for the purification of municipal sewage and some chemical wastes [7,10]. In the current literature and in industrial practice, because of its excellent resistance, low flammability, tunable properties and energy absorption capacity, polyurea has been employed in the field of ballistic coatings and blast protection to protect structures and buildings in recent years [2,3,4,5,6,8,13,14,15,16,17,18,19,20,21,22,23,24,25,26,27,28,29,30,31,32,33,34,35,36].

In early studies, the hardness and ductility of the polymer were critical properties when selecting for applications in protective coatings for blast and ballistic structures [37]. The current polyurea brands and mechanical properties applied in blast and impact protection studies are shown in Table 1. It should be noted that the mechanical properties of polyurea used for blast and impact protection are generally higher than 10 MPa, and the direction of research and development is to develop materials with high strength and high elongation.

Currently, it has been shown in numerous studies that polyurea is significantly strain rate sensitive (dependent), i.e., the mechanical properties of polyurea change noticeably when high strain rate loads are applied [24,37,41,42,43,44,45]. In addition, the mechanical properties exhibited during blast and impact loads are distinctly different from those under quasi-static conditions. This is also one of the critical features of polyurea in the blast and impact protection field. Therefore, the quasi-static mechanical properties can only be accepted as referential parameters in blast and impact protection and not as core parameters.

Based on the characteristic of blast and impact loading, polyurea applied in the protection field needs to meet the following requirements:

- The ratio of hard and soft segments of polyurea should be reasonably optimized to ensure that the material provides high strength while being able to resist large deformation, inhibit structural damage and reduce fragmentation rate.
- It is essential for polyurea to be thermally stable; therefore, the material can maintain high mechanical properties in response to high temperatures coupled with impact loading.
- The material should exhibit a high elastic modulus and long plastic stage when subjected to medium or high strain rate loading, as well as a high loss modulus and storage modulus, thus fully dissipating energy in the deformation process.
- The un-relaxed and relaxed modulus of the material should be increased as much as possible to absorb and dissipate wider frequency bands of loading.

In this paper, the research and applications of polyurea in the field of blast and impact protection are reviewed, including the optimization of materials, protection mechanism and protection performance of different substrates for blast and impact protection. The research direction and development trend of polyurea applications in the field of blast and impact protection are identified.

## 2. Materials Optimization

Polyurea exhibits high chemical stability and excellent physical properties. With the aim of providing blast and impact protection for different substrates in various environments, the ratio of hard and soft segments in polyurea needs to be adjusted by varying the content and category of isocyanate and amino compounds. Moreover, the incorporation of nano or micron particles also facilitates polyurea composites with better mechanical properties and higher blast mitigation performance.

### 2.1. Optimization of Polyurea

The soft segment of polyurea is composed of long polyether chains, forming amorphous domains, and the hard segment is composed of urea bonds, forming hard segment microphase domains connected by hydrogen bonds [10]. The hard segments are extensively hydrogen-bonded and serve as reversible physical crosslink and reinforcing components, thereby imparting excellent mechanical properties to the polymer, including the modulus, hardness and tear strength [1]. The soft segment, instead, offers flexibility, softness and low temperature resistance to the material [13]. The length of the soft segment affects the mechanical properties of polyurea under quasi-static and dynamic conditions. With the soft segment length increasing, the tensile strength decreases proportionally, and the glass transition temperature (Tg) decreases accordingly [1,46]. By the principle of time–temperature superposition (TTS), the decrease in Tg allows the material to maintain resilience against high strain rate loads (e.g., blast and shock loads) that are less susceptible to brittle damage. 

The urea bonds in the hard segment perform a very important role. It is expected that the loss spectrum can be enhanced through the control of the size, properties and distribution of hard domains in block copolymers [8]. A large number of studies on the thermal stability of polyurea show that polyurea degrades in a one-step behavior under high temperatures. Its decomposition starts with the decomposition of the urea bonds in the hard segment, and the decomposition temperature is in the range of 300–320 °C [6,47,48]. In cases where it is necessary to improve the thermal stability of polyurea, a combination of di- and tri-functional polyamines can be added to the formulation, which enhance the crosslinked structure of the material and thus improve its thermal stability [6]. 

**Figure 2:**
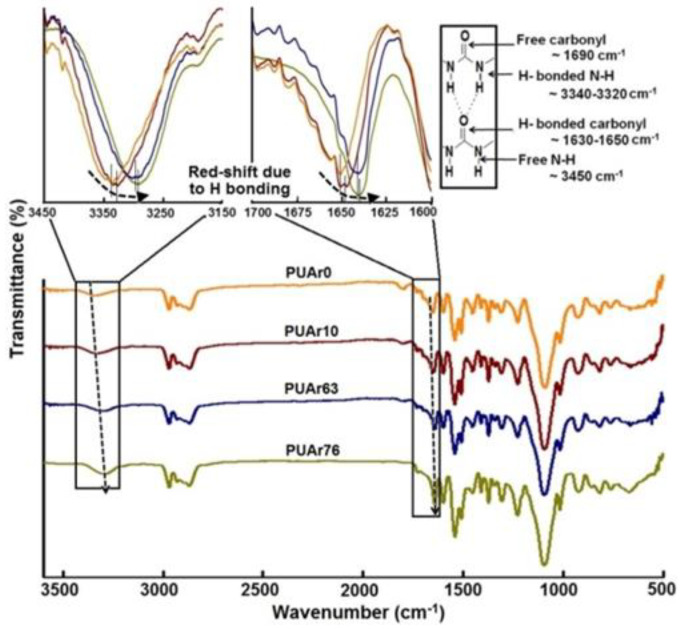
FTIR spectra of the red-shift associated with NH and C = O bands. Reproduced with permission from ref. [50].

Hydrogen bonds are one of the most significant molecular structural features of polyureas and play a crucial role in the morphology and mechanical behavior of polyurea [49]. The degree of hydrogen bonding can be approximately determined by the red-shift relevant to NH and C = O bands in the polymer [18,50], as shown in Figure 2. The storage modulus is in proportion to the degree of hydrogen bonds between the urea linkages in the polymer [50]. Although the storage modulus is also closely related to the mechanical properties of polyurea, the storage modulus and mechanical properties are also higher. With regard to mechanical properties, when the hydrogen bonds in the hard segments are broken, the mechanical properties of the material are reduced [2,50,51]. Li et al. [51] inferred that the mechanical properties of polyurea increase if hierarchical hydrogen bonding is introduced into the hard domains. Therefore, they replaced part of diisocyanate with a 2-Ureido-4[1H]-pyrimidone (Upy)-derived diisocyanate, which was a quadruple hydrogen bonding strategy to promote the mechanical properties of polyureas. Upy units enable energy dissipation more efficiently due to the breakage of stronger quadruple hydrogen bonds upon deformation [2,52]. 

For component A of polyurea, the increase in the isocyanate component means an increase in crosslinking density, which increases the storage modulus and makes polyurea more sensitive to temperature changes. Huang et al. [53] modified polyurea via the reaction of a polycarbodiimide-modified diphenylmethane diisocyanate and an amine-terminated polyether and found that the modulus and the loss factor increased with increasing frequency, but at elevated temperatures, the modulus was not rate dependent.

For component B, the crosslinker and chain extender play a vital part in determining the properties of the polymer. The crosslinker affects the physical and chemical crosslinking in the polyurea molecular structure. Tripathi et al. [45] found that polyurea exhibited relatively low tensile strength at a crosslinker content of 1.2 mol%, but the material showed high strain rate sensitivity. During medium or high strain rate loads, the material strength is enhanced significantly by a high strain rate sensitivity, a property that is extremely beneficial for blast and impact protection. The chain extender drives the phase separation process and influences the formation of hard segments [4]. The chain extender is introduced into the mixture of amino polyethers prior to the reaction and reacts with the isocyanate group in the prepolymer during the reaction to extend the chain, producing carbamate or urea bonds. Therefore, the content of the chain extender needs to be considered when calculating the hard segment content. The hard segment content is generally calculated as the ratio of the mass of the isocyanate and chain extender to the total mass, which is shown in Equation (1):

(1) $$\mathrm{Hard}\;\mathrm{Segment}\;\left(\%\right)=\frac{m_{\mathrm{iso}\;}+{\;m}_{\mathrm{ext}\;}}{m_{\mathrm{iso}\;}+{\;m}_{\mathrm{ext}\;}+{\;m}_{\mathrm{amine}\;}}\times 100$$

where m_iso_, m_ext_ and m_amine_ refer to the amount (g) of isocyanate, extender and amine, respectively.

However, in the absence of the chain extender, the following formula is used for its estimation.

(2) $$\mathrm{Hard}\;\mathrm{Segment}\;\left(\%\right)=\frac{m_{\mathrm{iso}\;}}{m_{\mathrm{iso}\;}+{\;m}_{\mathrm{amine}\;}}\times 100$$

where m_iso_ and m_amine_ refer to the amount (g) of isocyanate and amine, respectively.

**Figure 3:**
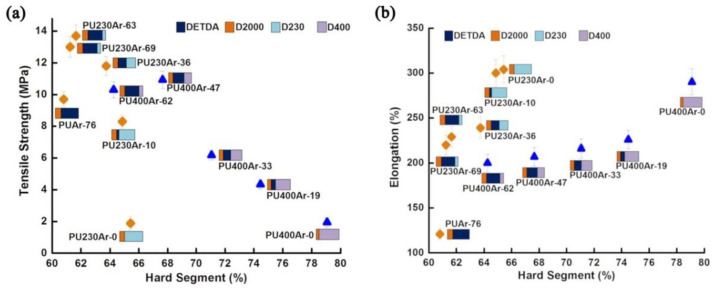
Influence of replacing DETDA with aliphatic chain extender on tensile strength (**a**) and elongation (**b**). The percent contribution of aromatic and aliphatic chain extender is also incorporated in the figure. Reproduced with permission from ref. [4].

With respect to the polyurea reaction, chain extenders increase the molecular weight of the material and lead to the formation of strong bonds between polymer segments, making it less likely that the bubbles formed during the polyurea reaction grow, thus improving the mechanical properties of the material [6]. The chain extender compels the formation of urea linkages close to each other, which in turn facilitates the formation of hydrogen bonds in the hard segments [4,50,54]. Tripathi et al. [4] further confirmed that adjusting the ratio of aromatic and aliphatic chain extenders allow the growing macromolecule to be arranged in an optimal hydrogen bonding pattern, resulting in improving the mechanical properties of the material. In the selection of chain extenders, by replacing a small number of aromatic chain extenders (e.g., diethyltoluenediamine (DETDA)) with low molecular weight aliphatic chain extenders (e.g., D230), the polyurea coatings exhibited better mechanical properties (as shown in Figure 3). Iqbal et al. [54] introduced aliphatic and aromatic chain extenders into the formulated amine co-reactants by the optimal ratio of the two forcing the growing macromolecules to orient in a manner that resulted in the formation of optimal hydrogen bonds. They found that the best mechanical properties of the material were achieved at 63% of the urea bond formed by the aromatic chain extender.

The polyurea spray construction process is one of the key attributes of polyurea that makes it widely used in various fields. Due to the fast curing characteristics of sprayed polyurea, the coating section shows a loose and porous cheese-like structural feature after the reaction film formation, which is favorable for absorbing energy under high-speed loads [36]. Controlling the reasonable matching of the viscosity of amines and isocyanates through the design of the polyurea formulation is an important part of optimizing the performance of polyurea spraying. Iqbal et al. [55] diluted isocyanate with propylene carbonate (10% *v*/*v*) and reduced the viscosity of the precursor from 85 to 48 mPa∗s (at 70 °C), which made the formulations effective for spraying and forming polyurea coatings with excellent mechanical properties. The long gel time can lead to the dripping of the polymer during the spraying process, which results in non-uniform films. Typically, formulations with a gel time of 60–180 s (rheological studies) are appropriate for spray processing applications [4]. The addition of aromatic and aliphatic chain extenders to polyurea formulations allows for the effective control of gel time and thus improves the processability of polyurea spray construction [18,50]. Recently, studies have found that aromatic chain extenders are much more reactive than aliphatic counterparts, which lead to noticeably short gel times [4]. For chain extenders, although they can adjust the gel times of polyurea, the mechanical properties reach a plateau over 15 days regardless of the type of chain extender contained in the formulation [56]. 

### 2.2. MD Simulation of Polyurea

The molecular design of polyurea formulations is required to obtain the polyurea with appropriate mechanical properties for a variety of applications. MD simulation is a significant method for optimizing the design of materials at the molecular level. With the rapid development of computational power in recent years, MD simulation has become more widely used in the design of materials [9,57,58,59]. 

**Figure 4:**
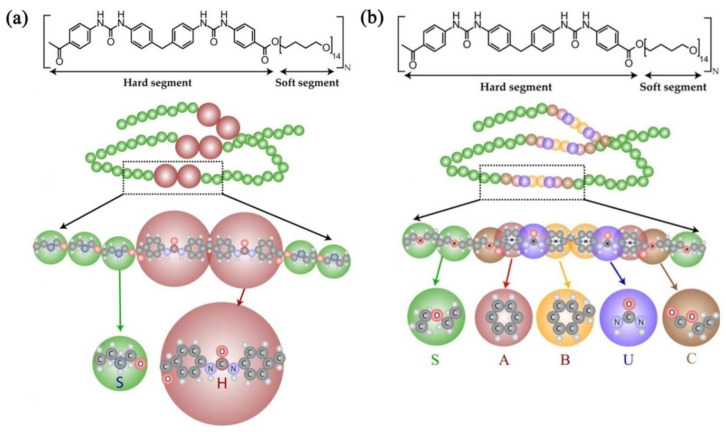
Polyurea CG models: (**a**) two-bead model and (**b**) five-bead model. Reproduced with permission from refs. [58,60].

Researchers mostly use coarse-grained (CG) models for polyurea molecular chains, but because early CG models are similar to the bead-spring model pioneered by Kremer and Grest, these CG models can only provide qualitative rather than quantitative predictions of the dynamic behavior of polyurea [58]. The CG models are not well-suited to represent large deformations, strong shock wave propagation or wide variations in temperature or pressure due to the lack of pressure and temperature transferability in the models. To address this problem, Agrawal et al. [58] handled each phenylmethane aminobenzoate and tetramethylene-oxide unit in the polyurea molecular chain with a single CG bead to form a two-bead model (Figure 4a). The parameters of the intra- and intermolecular force field of the CG model were obtained in a rigorous manner by the use of the iterative Boltzmann inversion (IBI) method and the introduction of a ratio based on the mean squared displacements of the atomic and CG oligomer systems of the time-dependent dynamic rescaling factor, thus enabling the quantitative prediction of stress relaxation on timescales beyond microseconds [58]. On this basis, Agrawal et al. [60] developed the discrete finer five-bead model (Figure 4b), a method that addresses the shortcomings of the two-bead model, where the chains pass through each other and violate topological constraints, and the predictions of the CG five-bead model were fairly well-matched by experimental measurements of the dynamic shear modulus of the benchmark polyurea by DMA and ultrasonic wave transmission. Liu et al. [61] proposed a CG model similar to the united atom model, using the IBI method and scaling functions for training, which significantly reduce the number of iterations required and extend the robustness and applicability of the IBI method to complex polymer systems. The length range predicted via the CG model established by Liu et al. [61] is surprisingly consistent with the length scales determined by X-ray scattering experiments. By means of CG-MD, Yao et al. [62] investigated the sources of the shock absorption and impact resistance of polyurea, finding that the soft phase can store more strain energy compared to the hard phase, whereas the hard phase dissipates plasticity through the structural disruption of hard segments and hydrogen bond dissociation.

Although the CG model significantly reduces the computational cost of the model with respect to the guaranteed time scale and the realization domain, additional empirical model parameters are introduced into the model in order to describe the interactions of the individual beads [57,58,60,61,63]. In contrast, all-atom MD simulations do not require additional empirical parameters, as they are based entirely on interatomic potentials. Using all-atom MD simulations, Heyden et al. [57] found that the segregation of polyurea hard chain segments into hard domains under quasi-static loading made no significant impact on the overall response of the composite. However, with respect to the dynamic mechanical properties, MD simulations by Heyden [57], Grujicic [63] and other researchers have revealed that multiphase segregation can be expected to have a strong influence on the dynamic properties of polyurea, and that the impact resistance of polyurea is determined by segmental dynamics rather than stoichiometric variations. Following the study of Heyden et al., Manav et al. [64] investigated the shock response of the hard and soft domains independently using MD simulations. They found that the hard domains exhibited less compression and a relatively smaller temperature rise compared to the soft domain at a given shock pressure [64].

Simulations of the MD at the atomic scale show that the soft phase of polyurea has hyperelastic behavior and that the hard phase has elastoplastic behavior [65]. This is consistent with the conclusion obtained from the CG-MD model developed by Yao et al. [62]. The protection against penetration processes is achieved by the interaction of hard and soft phases, whereas the performance and behavior of the protection against high-intensity indirect shocks (e.g., explosive loads) are mainly controlled by the hard phase [65]. 

### 2.3. Incorporation of Reinforcing Materials

The mechanical properties of polyurea can be tuned by choosing different variations of diamines and diisocyanates as well as by adding assorted nano-inclusions and micro-inclusions to create polyurea-based composites [5,66,67]. Nano-inclusions and micro-inclusions dispersed in the matrix can also improve the interfacial strength of the material, resulting in some enhancement [68]. The integration of high stiffness and high damping materials (e.g., polyurea) into one material system appears to be somewhat contradictory in the material design. Nonetheless, the combination of advantageous properties can be achieved through the reasonable compounding of materials with different characteristics to enhance the performance of polyurea in blast and impact protection. The interaction between different materials can also improve the energy absorption capacity of the material. Adding an appropriate amount of reinforcing material enables the polyurea to crack inside the coating and to deflect the cracks during the damage process, increasing the absorption of the fracture energy. A large amount of crazescan also prevents crack expansion, reduces crack generation and improves the toughness of the composite [68]. The enhancement of the strength and stiffness of the composite depends, to a large extent, on the quality of the interfacial bond between the reinforcement and the matrix [3]. Due to a mismatch between the mechanical properties of the materials, delamination between the reinforcement and the substrate often occurs at the interface or in the interphase region due to stress build-up. Large amounts of nano-reinforced and micron-reinforced materials can easily reduce the dispersion and lead to a stress concentration effect that can negatively affect the mechanical properties of polyurea [22,43,68,69,70]. 

**Figure 5:**
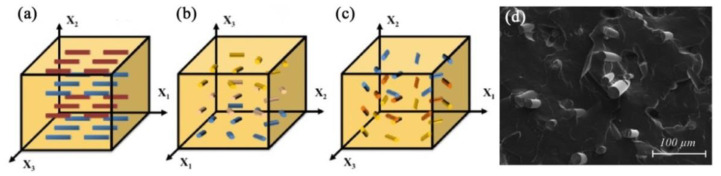
Models based on the periodic distribution of inclusions and SEM of PU-MG fractured surfaces: (**a**) Uniaxially oriented distribution, where fiber axis is always parallel to X_1_ axis; (**b**) In-plane oriented distribution, where fiber axis always lies on X_1_ X_2_-plane; (**c**) three-deminsional randomly oriented distribution; (**d**) SEM. Reproduced with permission from refs. [71,72].

Reinforcing fibers are widely adopted to improve the mechanical properties of polymers to achieve more load-carrying capacity [71,72,73]. High-strength and high-modulus polymer composites are produced by reinforcing the polymer matrix with high-strength and high-modulus fibers, the reinforcing fibers are employed to increase the strength and stiffness of the material, and the load is transferred to the fibers via the viscoelastic displacement of the matrix under stress [74]. The reinforcing fibers that are commonly used for enhancing performance are glass fiber (GF) [20,39,70,71,72,73,75], basalt fiber (BF) [22,23,76,77] and carbon fiber (CF) [78,79,80,81]. GF are of two types: chopped glass fiber and milled glass fiber (MG), of which MG is mostly used as reinforcing and filling media in plastic composites, adhesives and coatings. Nantasetphong et al. [72] added MG to create polyurea-milled glass composites (PU-MG). The dynamic performance of the PU-MG composite was studied by dynamic mechanical analysis (DMA) and ultrasonic wave measurements. Micromechanical models were created, and discussed micromechanical models were developed based on three different methods: (1) diluted random, (2) non-diluted random and (3) non-diluted periodic distributions of inclusions, as shown in Figure 5. Increasing the milled glass volume fraction drastically increased both the storage and loss modulus of the composites [72]. The glass microsphere polyurea composites exhibited a similar pattern, and due to the relatively low modulus of polyurea, there is a strong impedance mismatch between the glass spherical inclusions, resulting in considerable attenuation due to scattering [43].

Basalt-based products that are utilized in polymer modifications are mainly manufactured from BF as reinforcing agents for the polymer [23]. BF is a reinforcing material with low consumption in production and a higher performance than GF and has also been used by many researchers in recent years for polyurea reinforcement. Moreover, the adhesion properties of BF are higher than those of GF of equal size, giving a distinct advantage among fiber reinforcement materials [76]. Recent work by Barczewski et al. [22] has demonstrated that the addition of basalt-milled fiber (BMF) to polyurea can result in significant improvements in the mechanical and thermomechanical properties of the polyurea, with the crosslink density of the composite increasing almost fourfold at a filler content of 30 wt%. Basalt powder (BP) is an industrial waste material produced during the manufacturing of asphalt mixtures and track ballast that can be used as a filler in composites to improve the hardness and thermal stability of the material. Barczewski et al. [23] tried to use basalt powder instead of BF to reduce the cost of the product. With the aromatic polyurea spray system, the composites filled with a 20% mass fraction of BP showed the most favorable set of mechanical properties, with an increase of 76% in hardness, of 40% in tensile strength and in the thermal stability of the composites.

CF is one of the earliest fibers applied for composite reinforcement. CF-reinforced composites have many attractive properties, especially their strength, stiffness, light weight and toughness, and their applications have expanded to include aerospace fields, automotive structures, sporting goods and civil engineering. In addition, many academics have found that CF has a higher resistance to corrosion and blasting than polymer fibers at room temperature [59,82,83,84]. Two main forms of CF composites with polyurea are available: one based on CF interlayer composites and the other on milled or short-cut fiber composites with the same fiber reinforcement as other fiber reinforcement materials. In the case of the interlayer composite of CF with polyurea, this composite is known as CF-reinforced hybrid matrix composite (CHMC). Zhou et al. [78,82,85] found that CHMC has better protective properties compared to CF-reinforced epoxy (CF/epoxy), as evidenced by the fact that CHMC can maintain higher post-peak strength and good structural integrity with higher energy absorption during quasi-static extrusion. For milled or short-cut CF composite polyurea, due to the high process difficulty of fiber dispersion and the new high-performance fibers (e.g., multiwalled carbon nanotubes (MWCNTs)), few researchers have studied them systematically in recent years. 

MWCNTs are similar to graphene because they consist of single-atom-thick sheets of carbon atoms wrapped around each other to form tubes and are an emerging reinforcing material. Petre et al. [69] used functionalized MWCNT for the reinforcement of sprayed polyurea and found that the reaction between the isocyanate and the hydroxyl groups corresponding to the functionalized MWCNT formed a strong bond between the reinforcement and the polymer matrix, which can help to improve the mechanical properties of the composite. Petre et al. [86] investigated the protective capabilities of MWCNT-OH-polyurea, including its chemical, biological, radiological and nuclear (CBRN) attack protection properties. The results show that the composites provide more than the maximum protection time foreseen by the international standards (24 h) for a contamination density of 50 g/m^2^ of persistent chemical warfare agents (CWAs) and that reinforced polyurea can be used to manufacture individual and collective protection equipment against CWAs. Following this, Petre et al. [67] carried out experiments on the impact resistance of MWCNT-OH-polyurea, and the ballistic imprints, as a consequence of shooting, were reduced by more than 60%.

**Figure 6:**
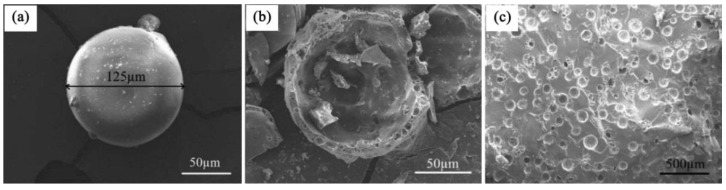
SEM images of fly ash (FA) and fly ash/polyurea (FA/PU) composites: (**a**) intact fly ash; (**b**) broken particles; (**c**) FA/PU composites. Reproduced with permission from ref. [27].

Fly ash (FA) is composed of aluminum oxide (Al_2_O_3_) and silicon dioxide (SiO_2_) and features a hollow sphere structure with a porous shell, as shown in Figure 6. This structure gives the material many excellent properties, such as low density, excellent damping and energy absorption properties, which are important for improving the performance of composite materials. Qiao et al. [27] investigated the influence of FA volume fraction on the dynamic mechanical behavior of FA/PU. They found that the storage modulus increases rapidly with the increase in FA volume fraction and that the relative modulus values jumped in the glass transition region, which may be due to changes in particle-to-particle contact interactions. At higher FA volume fractions, Tg shifts to a higher temperature, which may indicate a reduced response to the contribution of the viscoelasticity of the polymer matrix and enhanced crosslinking. 

The relaxation behavior of polyurea is attributed to the chain kinematics of the polyurea molecules and the variation in chain length and angle [87]. The relaxation of polymers has an effect on their mechanical properties, in particular, on deformation, yield, fracture behavior, toughness, impact strength and brittle–tough transitioning [88]. Recent work by Qiao et al. [87] has demonstrated that stress relaxation in FA/PU at high temperatures is caused primarily by the debonding of the ash hollow spheres from the polyurea matrix, whereas the relative displacement between the contacting ash hollow spheres dominates the stress relaxation in the material at low temperatures. The pristine zirconia particles in the monoclinic phase can also improve the relaxation spreading of polyurea and the relative intensity of the loss factor in DMA, and the surface functionalization can be omitted, since oligomeric diamine can effectively facilitate the dispersion of zirconia particles [21].

**Figure 7:**
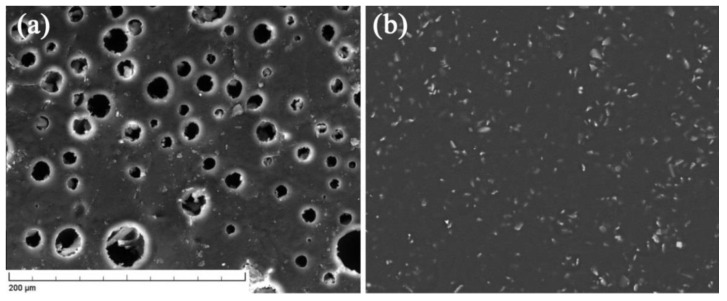
SEM images of (**a**) neat polyurea and (**b**) polyurea reinforced with irregular alumina particles. Reproduced with permission from ref. [74].

Alumina particles are another reinforcing filler for polyurea. Oddy et al. [74] added irregular alumina particles to the polyurea as a reinforcing material and found by SEM that the addition of alumina particles appeared to inhibit the tendency to entrain and trap air in the polyurea during the spraying process, achieving the elimination or reduction in porosity in the pure sample. Figure 7 shows SEM images of the middle plane of the neat polyurea sample and the alumina-reinforced polyurea sample.

In addition, researchers have recently found that adding reinforcing materials to polyurea increases the strain rate sensitivity of polyurea. Liu et al. [68] revealed that the flow stress and compressive strength increased by 12.8% and 12.1%, respectively, under a strain rate of 8000 s^−1^ load compared to unreinforced polyurea with the SiC-reinforced polyurea.

## 3. Protection Mechanisms

**Scheme 2:**
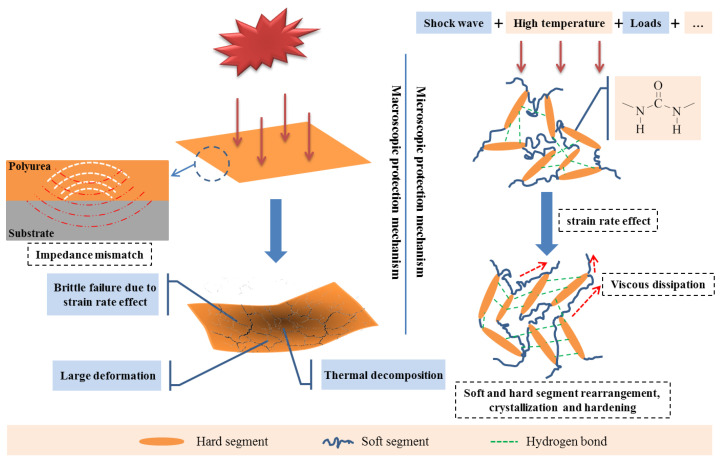
Schematic illustration of the protection mechanisms of polyurea coatings.

With blast and impact loading, the effect of polyurea on the dynamic response of the protected substrate is complicated. Both Liu [40] and Iqbal et al. [54] considered that shock-wave-induced hard domain ordering and crystallization, rearrangements and neutralization appear to be the dominant modes of energy dissipation in polyurea under blast loading conditions. This process is also accompanied by viscoelastic dissipation of the material, enhanced mechanical properties due to strain rate effects and the effect of impedance mismatch between the substrate and polyurea. The blast and impact protection mechanisms are shown in Scheme 2.

### 3.1. Soft and Hard Segment Rearrangement, Crystallization and Hardening 

The high polarity of the hydrogen and oxygen atoms in the urea groups in the absence of loading facilitates the formation of hydrogen bonds between urea linkages, and the resulting copolymers show a hard-domain structural morphology characterized by high Tg embedded in a soft viscoelastic matrix with low Tg. For aromatic polyurea, hydrogen bonding also can be synergistically combined with π–π stacking interactions between the aromatic rings to promote the microphase separation of polyurea [57]. This particular structure constitutes an important mechanism in the impact protection of polyurea. 

Under explosive loading, the material is subjected to the coupling effect of temperature and loading [2,17]. The disruption of the hydrogen bonds is affected by temperature in two stages, namely, slight dissociation and dramatic destruction corresponding to the coarsening process [2]. Due to the change in hydrogen bonds, it follows that the soft and hard segments in polyurea are structurally rearranged. In the range of 85–165 °C, dissociation of the bidentate hydrogen bonding occurs, the connection between the hard segments is lost and the microstructure of the material changes insignificantly. However, the loss factor decreases significantly, and the yield point of the material is missing. The hydrogen bonding begins to deconstruct dramatically from 165 °C, and the coarsening process begins to occur. The hard segments begin to self-assemble, the width and length of the band morphology become larger with increasing temperatures and the physical crosslinking density in the system decreases. The coarsening process promotes a microstructural transformation. In the second stage, the strength of the polyurea as well as the Young’s modulus undergoes a significant decrease. As the temperature increases, polyurea undergoes thermal decomposition. Lyu et al. [13] observed by thermogravimetric experiments that the thermal decomposition rate of polyurea reached the maximum at a temperature of 386 °C. At a temperature of 700 °C, the polyurea decomposed into carbon residue and lost its protective properties [13]. By comparing the FTIR spectra of polyurea before and after the explosion, Zhang et al. [36] found that the area of the carbonyl stretching region and imino stretching region was decreased. It was shown from the practical explosion data that the hydrogen bonds in polyurea were broken, and the microphase separation was also damaged after subjecting the polyurea to the explosion load.

In the process of the deformation of polyurea subjected to blast and impact loading, unlike under static loading, where cracks develop and propagate along weak zones (soft segments), the response of the hard segments of polyurea can more easily be activated, and material cracks propagate along with hard segments in the microstructure [44]. The hard segments tend to be ordered in the molecular structure in polyurea, and this tight arrangement of polar chain segments in turn promotes the formation of hydrogen bonds and the increased crystallization of the material. This also leads to the strain rate sensitivity (dependence) of polyurea [44]. Moreover, this process is accompanied by the breakage and reorganization of hydrogen bonds. The intermolecular hydrogen bonding state plays a critical role in dynamic hardening and strengthening [89].

Regarding the loading frequency, the stress caused by the blast or impact exhibits wide-ranging frequency components, and the optimal dissipative material is one in which the frequency of the stress wave matches the critical frequency and in which the optimal damping of the material matches the frequency [25,46]. It is usually associated with explosive loads of 400–500 Hz [38]. Iqbal et al. [54] found that their synthesized polyurea required at least 1013 Hz for the glass transition to occur at ambient temperatures, which is not enough to allow the glass transition of polyurea. The same phenomenon was found in concrete structures protected by polyurea by Liu et al. [40]. This indicates that blast loading puts the polyurea in a strain-hardened state when only the loading frequency is considered, which is not sufficient to induce several of the above polyureas to enter the glass state and undergo brittle failure.

### 3.2. Viscous Dissipation within the Material and Strain Rate Effects

In tension and compression experiments, polyurea has a significant nonlinear stress–strain relationship [16,24,37,38,45,56,90,91]. It is a typical viscoelastic material, which exhibits viscoelastic properties with respect to viscous dissipation to absorb impact energy and strain rate effects in its mechanical properties. 

During material deformation, the soft segments connected in the material allow the hard segments to remain covalently connected to each other. The viscous dissipation of polyurea occurs mainly in the soft segments, with energy dissipation occurring through chain movement [92]. Perturbation by straining amplifies the strain energy by the phase separation morphology of the material. Through the development of a hybrid all-atom/coarse-grain (AA/CG) model, Zheng et al. [93] found that a strong correlation is created between the polyurea hard segments through hydrogen bonding, which reduces the mobility of the hard segments and leads to a substantial difference in mobility between the hard and soft segments. The difference in mobility causes the relative displacement of the polyurea molecular structure during deformation, which results in energy consumption by means of internal friction. High-frequency stress waves traveling through the viscoelastic material undergo multiple load–unload cycles, which can lead to a large amount of energy dissipation in a short period of time [25]. The process of energy dissipation is accompanied by an increase in temperature, which in turn affects the mechanical response [19,46,94]. Liu et al. [68] found that impact energy acting on polyurea is transformed into thermal energy under a high strain rate impact, resulting in thermal softening and stress reduction. Mott et al. [19] employed high-speed thermography to measure transient temperature changes in the deformed polyurea, and they found that the unusual viscoelasticity degree is due to a high Tg and large internal friction. The coupling causes the hard domains to move resonantly during shock propagation, thereby enhancing the energy dissipation [57]. The energy is absorbed by the resonance between the polymer segmental dynamics and the impact frequency. For higher frequencies, the hard domains become a resonator and consequently contribute to increase the overall material loss [8]. Moreover, as a result of the coating transient hardening, the reversible change in the material to a glassy state increased the modulus by about three orders of magnitude, which caused lateral spreading of the impact force, reducing the impact pressure [57,95,96].

**Figure 8:**
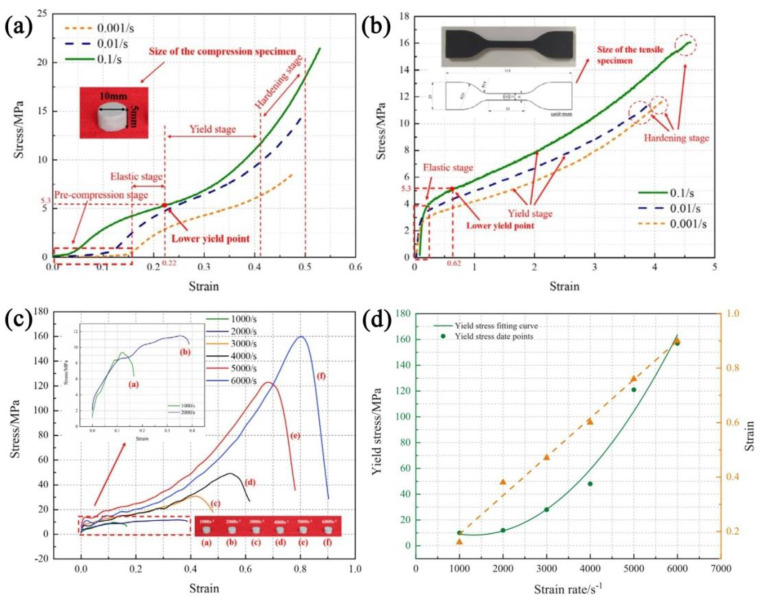
Mechanical performance of AP103-type polyurea: (**a**) quasi-static compression, (**b**) quasi-static tensile, (**c**) dynamic compression and (**d**) corresponding yield stress and strain. Reproduced with permission from ref. [16].

The strain rate sensitivity (dependence) of polyurea contributes to its use in structural blast and impact resistance. The marked sensitivity of the strain rate, i.e., a shift from rubbery behavior towards glassy-like behavior upon high strain rate deformation, can be exploited to obtain substantial energy dissipation under particular conditions [20,37,89,96,97]. The strain rate sensitivity is closely related to the glass transition of the material [40]. In the study of Wu et al. [16], it was found that polyurea exhibits rubbery behavior at low strain rates and glassy behavior at high strain rates at room temperature. This is consistent with the dynamic thermo-mechanical properties of polyurea at different temperatures, which is fully in accordance with the law of the TTS. For the mechanical properties, Wu et al. [16] found that at high strain rates, unlike superelasticity at low strain rates, polyureas exhibit a significant yield slip, strain hardening properties and more pronounced strain rate effects (Figure 8). It can be observed from Figure 8d that the yield stress of polyurea increases continuously and exponentially with the increase in strain rate. This is in agreement with Giller et al. [96], who suggested that the strain rate sensitivity of polyurea is greatest in its glass transition region. Gamache et al. [98] found that, in laminate structures, the mechanical stiffness of the coating is increased due to the strain hardening of the polyurea, which can laterally disperse the impact forces. This process allows for the additional work of deforming larger areas to enhance ballistic performance [98].

### 3.3. Impedance Mismatch between Substrate and Polyurea

**Figure 9:**
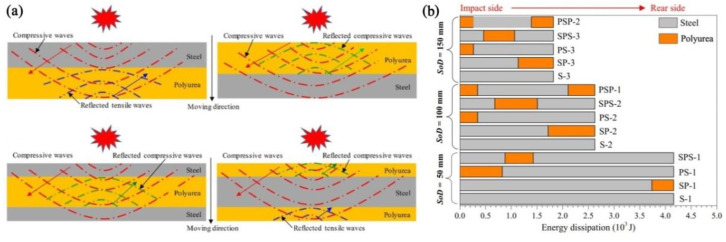
(**a**) Schematic of stress wave propagation in different structures and (**b**) the energy dissipation of each layer. Reproduced with permission from ref. [34].

The impedance mismatch between polyurea and the substrate during stress wave propagation is a major reason for the blast and impact resistance of polyurea from a macroscopic point of view [25,33,34]. Hailiang et al. [34] analyzed the propagation of stress waves in polyurea-coated steel plates with different structures and the energy dissipation mechanism through experimental and numerical methods. The propagation path of stress waves in composite plates was characterized based on the damage phenomena of the composite structures (Figure 9a). They quantified the energy dissipation of composite plates with different structures at equal total area densities (Figure 9b).

Wu et al. [16] also found the effect of impedance mismatch between polyurea and the substrate material on the blast resistance performance, and they found that, if the polyurea layer is not firmly bonded, the inertial deformation caused by polyurea at the interface joint makes the two separate, weakening the unloading effect of the reflected unloading wave and increasing the deformation deflection. With the polyurea located on the blast surface, polyurea is subjected to blast loading that causes a tensile wave, which results in bulge deformation, as described in ref. [15]. 

## 4. Research and Application of Polyurea in Blast/Impact Protection

Polyurea is a new protective material that has attracted a large number of researchers in different protective fields to study its characteristics [16,38,54,99,100,101,102,103,104,105,106,107]. According to the protection substrate types, the current research of polyurea in blast and impact protection can be divided into the protection of civil engineering structures, the protection of metal structures and protective applications for composite materials.

### 4.1. Protection of Civil Engineering Structures

The majority of existing building structures are not designed at the beginning to survive the effects of blast loads. Civil engineering structures are brittle, with low flexural strength, and are basically unable to absorb strain energy in the absence of reinforcement. Once subjected to the blast load, the effects are extremely serious. Most casualties in civil engineering structures during external explosions are caused by structural disintegration and debris resulting from the explosion [39,73,108,109,110,111,112,113]. Polyurea is highly applicable in structural reinforcement due to its special mechanical properties. 

**Figure 10:**
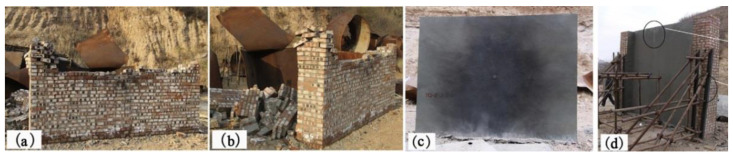
Comparison of unreinforced and reinforced masonry wall: (**a**) front of unreinforced masonry wall; (**b**) side of unreinforced masonry wall; (**c**) front of polyurea-reinforced masonry wall; (**d**) side of the polyurea-reinforced masonry wall. Reproduced with permission from ref. [108].

Wang et al. [108] found that sprayed polyurea can increase the ultimate blast resistance of clay masonry walls by a factor of 4.5–11, and the polyurea does not debond under the action of 8 kg TNT at a blast distance of 1 m (Figure 10). For gas explosion resistance in masonry walls, Gu et al. [110] studied masonry walls with polyurea sprayed on the front, back and both sides. It was found that the blast resistance of the front-side sprayed polyurea walls showed no significant increase, and the debris was still emitted from the backside of the walls. The blast resistance of the double-sided sprayed polyurea was optimal. Gu et al. [110] concluded that rear reinforcement is an acceptable option in the case of limited construction conditions.

For concrete protection, Shi et al. [39] compounded polyurea with woven glass fiber (WGF) based on the characteristics of polyurea and WGF. They found that the failure mode of the coating was changed from shear punching failure to tensile failure and caused a trend from local burst-induced perforation to overall plastic yielding of the reinforced concrete (RC) slab. Recently, Lyu et al. [13] proposed that the ability of polyurea-protected RC slab to maintain good integrity and stability was achieved by the combined effect of front and back blast surface coatings, i.e., the front surface coating resisted the high temperature generated by the blast and reflected the shock waves, and the back surface weakened the impact tensile waves and completely covered the concrete fragments. Through numerical simulations, they also demonstrated that polyurea coating increased the kinetic energy conversion rate of an RC slab, thereby improving the blast resistance of the RC slab [13].

Song et al. [73] used MG as reinforcing fibers, which were mixed with prepolymers and were sprayed onto the surface of the concrete beams along with the hardener to create the glass-fiber-reinforced polyurea (GFRPU). It was found that the GFRPU reinforcement can prevent the sudden spalling and debonding of concrete, and it retains the shear resistance capacity. 

**Figure 11:**
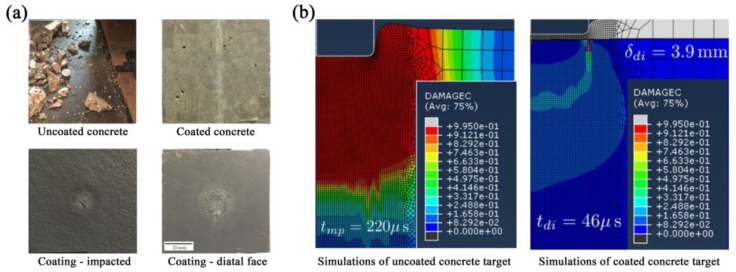
(**a**) Photographs and (**b**) simulations of the test specimens for a projectile impact velocity of 100 m·s^−1^. Reproduced with permission from ref. [109].

Fallon et al. [109] used a gas gun apparatus to conduct experiments on specimens with projectile velocities in the range of 45–150 m·s^−1^. It was found that the polyurea coating significantly reduced the impact damage of concrete through the tests and simulations (as shown in Figure 11) of Fallon et al. [109], and the coating was found to have the effect of delocalizing the damage. Nevertheless, the study by Fallon et al. did not consider the role of adhesion between the polyurea and the substrate, and the polyurea coating they used was pre-sprayed and pasted on the impact surface of the specimen. In contrast, the adhesion between the coating and the substrate is considered by many researchers to be an important indicator in the blast and impact resistance of polyurea [34,77,110]. 

For autoclaved aerated concrete (AAC), Chen et al. [104] employed carbon-fiber-reinforced plastics (CFRP) and polyurea to provide a total thickness of 4 mm polyurea coating on the top, bottom and sides of AAC. It was shown that, although the ultimate loads of polyurea-coated AAC slabs under quasi-static loading were slightly lower than those of slabs reinforced with CFRP, the bottom and double-sided polyurea-coated AAC slabs were much more resistant to blasts than CFRP-reinforced AAC slabs. It was also found that polyurea can hardly improve the static load carrying capacity of reinforced concrete arches in the study by Liu et al. [40]. Notably, in the study by Sonoda et al. [114], they revealed that the polyurea protective coating is also not effective against RC structures for the first impact loading action at low and medium strain rates. With repeated impacts, however, the improvement of polyurea on the impact resistance of RC structures is significant and can significantly inhibit structural cracking and rigidity loss [114]. Among the many researchers studying the polyurea enhancement of concrete structures against blast and impact loads, the polyurea inhibition of structural crack evolution and spalling is considered an important macroscopic level of protection. It was also found by Wu et al. [113] in experimental and numerical simulations that the improved protection performance of polyurea becomes more prominent at smaller scale distances. Regarding the location of coating protection, same as the masonry wall, the backside of the blast side coated with polyurea has been found to be the most effective protection measure in concrete blast protection [104].

Although it is obvious that polyurea enhances the blast and impact resistance of concrete structures, the design thickness of polyurea coating needs to be synthetically designed considering the actual engineering needs and the economics of structural reinforcement. Wu et al. [113] noticed that the maximum mid-span displacement of RC slabs decreased significantly when the thickness of the polyurea layer increased from 1 mm to 6 mm, whereas the slab deformation was less affected when the thickness exceeded 6 mm. Therefore, Wu et al. [113] suggested that the valid thickness of polyurea for RC slabs should be approximately 1–6 mm.

### 4.2. Protection of Metal Structures

With regard to the protection of metal structures, a large number of studies have now shown that polyurea provides better protection for airborne explosions when the coating is sprayed on the backside of the metal structure [16,26,33,34,105,115]. The polyurea coating on the backside dissipates energy by tensile deformation and reduces the impact of shock waves on the metal structure, inhibiting structural deformation. On this basis, Zhang et al. [36] revealed that, under the combined effect of blast shock waves and fragmentation, the damage to the steel plate is reduced despite the fracture damage of the high-hardness polyurea on the backside. Chen [32] and Li et al. [33] found that full-area spraying on the backside offers far better protection than partial spraying because of the confined polyurea boundaries, which can absorb energy through tensile deformation and the peeling of the coating. Jiang et al. [26] found that the polyurea layer inside the metal tank can provide an additional bending moment to the structure to suppress the deformation during external blast loading. In a recent study by Jiang et al. [116] on the equilibrium equation of the tank, the polyurea coating increased the area density of the tank, thereby reducing the displacement of the tank under blast loading. Moreover, for the thickness and location of the coating, Jiang et al. [116] concluded that, when the total thickness of the polyurea is the same, it is more efficient to spray all the polyurea on one side than to spray the polyurea on both sides of the tank. The mass of the polyurea coating also has some influence on the blast resistance performance of the metal tank, as it increases the inertial force on the loaded surface [26]. Furthermore, Yongqing [33] and Hailiang et al. [34] suggested that the main energy-absorbing components during the action of the blast load are the metal plate in the front. However, Yongqing et al. [33] found that, when in contact with polyurea coating steel plates, the polyurea coating absorbs more kinetic energy than the steel plates.

The coating located on the blast/impact surface has no significant protection against the deformation of the structure. Remennikov et al. [101] revealed that the excessively high heat generated by the blast causes the polyurea layer to melt near the center of the steel plate, which may affect the overall effectiveness of the coating in reducing the deformation response of the steel plate to approaching blast loads. Most of the early studies (e.g., ref. [117]) thickened the polyurea coating to improve protection. Nevertheless, when influenced by the impact load, the impact surface polyurea can reduce the stress concentration of the steel plate, thus delaying or preventing the fracture of the plate [118].

**Figure 12:**
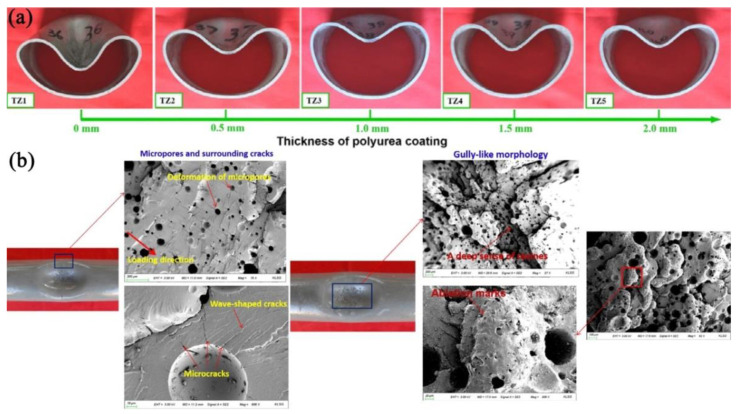
Damage of tubes subjected to blast loading: (**a**) Cross-section damage and (**b**) micro-morphology of polyurea layer. Reproduced with permission from ref. [16].

However, Wu et al. [16] found that the protective performance of polyurea coating on the blast-face cannot be underestimated. The deformation of the circular tube protected by polyurea on the blast surface decreases and becomes progressively smaller as the coating thickness increases (Figure 12a). By analyzing the microscopic morphology of the blast surface (Figure 12b), Wu et al. [16] concluded that high temperature resistance and flame retardancy are essential properties of polyurea against blast loads. It was concluded by Zhang et al. [36] that the severe ablation of the blast surface by the detonation products substantially reduce the energy absorption efficiency of the coating. Comparing the energy absorption efficiency of the coating on the blast side and the back blast side, it is undeniable that the energy absorption efficiency of the coating on the blast side is lower. 

Furthermore, similar to the blast protection of concrete structures by polyurea, it is of importance to have adhesion between polyurea and metal substrates. Rijensky et al. [119] revealed through simulations that the strength characteristics of the primer used prior to this coating should be considered in blast resistance protection. Premature cracking of the polyurea after the failure of adhesion to the substrate limits the effect of the polyurea on energy absorption [36].

With respect to reducing the impact of the explosion on the external environment, Matos et al. [99] used a combination of a semi-spherical pressure vessel and digital image correlation (DIC) to simulate the underwater implosion of polyurea-protected aluminum tubes and showed that the exterior and interior coated tubes release less energy after the implosion than the uncoated tube, with the interior coated releasing the least energy, and that doubling the volume of the coating does not significantly improve the mitigation effect. Subsequently, they also found that the polyurea causes a longer delay in the implosion pulse with this set of devices, which is sufficiently large to reduce the peak energy values by 35% for external coatings and 50% for internal coatings [120]. Remarkably, owing to the peculiarities of the fluid–solid coupling response to the underwater explosion (UNDEX), the protection and penetration mechanisms of metal structures coated with polyurea under the loads of UNDEX are vastly different from those of airborne explosions [115]. Li et al. [115] found that the aluminum plate coated with polyurea on the frontside ended up with the least severe plastic deformation when the underwater shock wave impacted, whereas the aluminum plate coated with polyurea on both sides showed the largest severe plastic deformation, which is contrary to the protection law of polyurea in the case of the airborne explosion.

**Figure 13:**
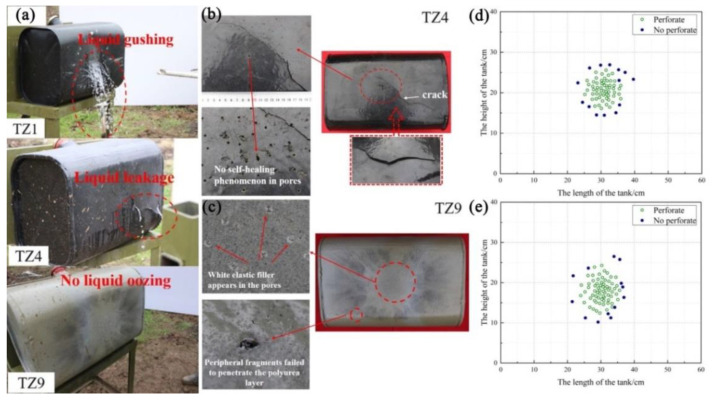
The damage of the tank after the combined action of shock waves and fragments: (**a**) Water seepage situation of the tank; Blast surface damage of (**b**) TZ4 and (**c**) TZ9; Fragment distribution of (**d**) TZ4 and (**e**) TZ9. The TZ1, TZ4 and TZ9 are the tanks without polyurea protection; 6 mm AMMT-53 polyurea-protected tank and 6 mm AMMT-55 polyurea-protected tank, respectively. Reproduced with permission from ref. [15].

Penetration is a special impact process with a high temperature, high pressure and high strain rate. Polyurea has been found to sharply increase the ballistic limit of the underlying steel substrates, with one significant mechanism being the impact-induced rubber-to-glass state transition [14,121]. Recently, polyurea was found to cause reductions in the residual velocity of the projectiles and to increase the ballistic performance of metal plates [14,92,122]. Metal plates coated on the frontside showed a more pronounced increase in ballistic limit and specific energy absorption than the backside [14]. Zhang et al. [14] also noticed a decrease in the total ballistic performance of the plates when the backside was sprayed with high-hardness polyurea. However, in early studies, researchers found that polyurea has no benefit in increasing the ballistic limit of steel plates [123]. This may be a result of the choice of rigid polyurea as a protective coating for the purpose of improving the strength of the composite structure. The coating underwent brittle damage at the time of penetration. However, for preventing the penetration of fragments, rigid polyurea such as AMMT-53 mentioned in ref. [15], although prone to cracking, local crushing and local collapse (Figure 13b,d), provides a strong barrier to fragments due to its high hardness, which can effectively reduce the perforation rate.

With high strength and high break elongation, the position of the polyurea has a lesser effect on the protection, and the effect of back spraying is not higher than 20% compared to the front protection effect [35]. In this case, the underlying substrate maintains adequate bending stiffness to allow impact-induced polymer transition, which, in combination with the rupture and dissipation of the pressure wave due to impedance mismatch, contributes to large increases in ballistic penetration resistance [98,121]. Similar to the blast protection mechanism, it is also important that bonding strength contributes to the penetration resistance of polyurea/steel composite plates. Sun et al. [35] found that, if the bond strength between polyurea and the steel plate is enhanced, more of the kinetic energy of the projectile is converted into other forms during the penetration process. In laminate structures, the number of the layers of the laminate structure and the nature of the metal components were found to have very little effect on performance, which differs from blast/impact protection [98].

From the micro-protection scale, MD simulations of multilayer aluminum–polyurea nanostructures reported by Dewapriya et al. [122] have revealed that both the ballistic limiting velocity (V50) and specific penetration energy of the multilayer material and the aluminum nanofilms are remarkably higher than the experimental measurements for any material. In a further study, with an impact velocity of 1 km/s, the specific penetration energy of the polyurea–metal bilayers (5.39 MJ/kg) was found to be remarkably higher than the value reported for any nanomaterial at a similar velocity (i.e., 3.8 MJ/kg) [124]. Nevertheless, MD simulations at the nanoscale by Dewapriya et al. [125] showed that the polyurea at the impact face is more effective in mitigating the impact-induced damage, which is a result contrary to that obtained from experiments performed at the macroscale.

Moreover, since polyurea has a strong self-sealing property, it has the unique advantage of preventing the leakage of special containers such as water tanks and oil tanks from being penetrated [7,15]. Huang suggests that the self-sealing property is due to the high modulus of elasticity of polyurea, which results in the coating closing on itself after the coating suffers penetration damage [7]. However, not all polyureas exhibit this property, and rigid polyurea can only lead to less leakage, as shown in Figure 13a. Wu et al. [15] attributed this self-sealing property of polyurea to the thermal effect of high-speed fragments penetrating the polyurea layer to produce a grayish-white elastic filler (Figure 13c) to form the self-healing effect.

### 4.3. Protective Applications for Composite Materials

Given the mechanical performance characteristics and limitations of polyurea with respect to mechanical strength, numerous scholars have used polyurea in the field of composite impacts and blast and penetration resistance to reinforce the protective structure and have achieved relatively obvious protection. Tekalur et al. [38] found that combining polyurea as an interlayer material for E-glass vinylester (EVE) materials can increase the blast resistance of EVE by 100% for air blast loading. Leblanc et al. [28] demonstrated that the transient response of polyurea-coated E-glass plates improve with increasing coating thickness for UNDEX loading through conical impact tubes combined with DIC and simulations, with the polyurea coating located on the backside of the plates providing better performance than that located on the loading surface. In their further study, they found that both double thickness plates and coated plates of the same thickness (1.524 mm) are superior to single layer (0.762 mm) E-glass plates, and that the polyurea coating is superior to the thicker uncoated plate with respect to reducing material damage [29]. The damage of the coated composites decrease dramatically with increasing coating thickness compared to the baseline cylinders [30]. In the finite element simulation of the EVE-matrix-reinforced composite panel, Phuong et al. [126] developed a finite element model of polyurea-enhanced composite (PEC) panels and used the Mooney–Rivlin hyperelastic law to simulate the behavior of polyurea. They found that the polyurea coating applied to the backside of the panels improves the delamination problem of EVE multilayer panels, and both the fiber and the matrix are reduced.

However, the effect of polyurea on reducing delamination in multilayer panel structures is not applicable to this material. In multilayer fiber/vinylester composite panels, the polyurea coating as a backing layer improves the fiber damage resistance when subjected to blast loads, although it does not play a significant role in reducing interlayer delamination [127].

**Figure 14:**
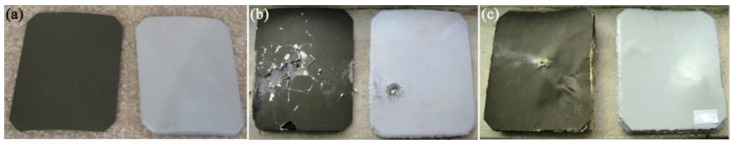
(**a**) Standard ballistic protection plate and MWCNT-OH-reinforced polyurea-coated ballistic protection plate before shooting; (**b**) exposed side after the shot; (**c**) reverse side after the shot. Reproduced with permission from ref. [69].

Polyurea composites against penetration are a hot research topic for non-metallic penetration-resistant materials in recent years. The penetration resistance tests reported by Petre [69] for MWCNT-OH-reinforced polyurea revealed that the reinforced composite coated protective ballistic plate was not penetrated, and it provided III-A protection against. 44 Magnum Semi Jacketed Hollow Point (SJHP) ammunition, whereas the standard ballistic protection plate was perforated on a single impact (as shown in Figure 14). Liu et al. [128] analyzed the morphological characteristics of polyurea CFRP composite panels after penetration damage in detail and summarized four points of failure modes, namely, (1) compression shear failure on the frontside of polyurea, (2) tension shear failure on the backside of polyurea, (3) petaling failure and (4) spallation and punching perforation failure. By comparing the residual velocity, energy absorption ratio, deformation and damage level of specimens at various coating locations, Liu et al. [128] concluded that spraying polyurea on the backside of CFRP composites can significantly improve the ballistic performance of composite panels, and backside spraying amplifies the damage area.

Multilayer polyurea/ceramic nanocomposite is a new protective material made through polyurea composite with silicon carbide (SiC), which has a high ballistic limit velocity and specific penetration energy. To accurately model the non-bonded interactions of the polyurea/SiC interface, Dewapriya et al. [129] used density functional theory (DFT) calculations to obtain an accurate set of Lennard–Jones parameters and found that the interfacial adhesion between the polymer and the ceramic is relatively weak. Subsequent MD simulations by Dewapriya et al. [129] showed that multilayer ballistic performance is significantly affected by interfacial adhesion.

Numerous scholars sought to improve existing helmets (e.g., advanced combat helmets (ACH)) by exploiting the shock-mitigation ability of polyurea to reduce traumatic brain injury (TBI) caused by intra-cranial cavity [130,131,132]. According to the simulations of Grujicic et al. [130,131], polyurea was found to significantly lower the peak load experienced by the brain, and these improvements in the helmet shock-mitigation performance were obtained only in the case of helmet-polyurea-based internal lining through the study of polyurea coating location. In considering the explosive charge standoff distance and the TBI-inducing intra-cranial metric, it is beneficial to spray polyurea on the exterior of the helmet [132].

## 5. Conclusions and Outlook

Polyurea, as a new functional material, has been rapidly promoted to various protection fields such as vehicle wear resistance, building waterproofing and structural corrosion protection. Over recent years, with performance beyond that of conventional coatings, including tensile/compression strength, breaking elongation, tear strength, strain rate sensitivity and energy absorption properties, it is sufficient for enhancing the stability of various structures and materials under dynamic loading, which makes polyurea a potential alternative to the currently used materials for reinforcing existing structures. 

As mentioned earlier, with the new material design and new raw materials, there have been significant improvements in all aspects of polyurea performance. However, there are some disadvantages in the application of polyurea, and its main disadvantages include:

- Extremely high demands are placed on the spraying process, and in the event of uneven mixing, the completed sprayed surface needs to be completely removed.
- The repair of damaged coatings is quite difficult and requires cutting and surface treatment of the original coating.
- The cost of spraying polyurea (material loss, equipment cost, etc.) is high, and the current spraying technology is suitable for continuous operation of a large area, whereas for small-area spraying, the loss of raw materials is relatively large for each spraying.

Although the above disadvantages have some effects on the application of polyurea in practical protection works, considering the protection effect of the material, the advantages of polyurea in blast and impact protection are significant compared with polyurethane, epoxy resin and other materials.

Numerous studies on the protective properties of polyurea are currently focused on how the material can be used to retrofit existing structures, addressing the characteristics of each area of application. It requires researchers to redesign the material formulation and to adapt the polyurea for different application environments. In structures exposed to the outdoors for long periods of time, for example, ultraviolet (UV) radiation is higher in energy than the activation energy of the polyurea, causing changes to the segmental microstructure, which accelerate the aging of the polyurea and cause damage to the mechanical and physical properties [10,133]. The UV aging resistance of polyurea is required to ensure its protection during its service life. Therefore, it is not rigorous to simply equate the various types of polyurea. Based on recent studies, with respect to the material reaction principle and the spraying process of protective coating, ordinary polyurea is almost the same as that used for blast and impact protection polyurea. 

The protection of existing structures is due to the inability of existing structures to withstand the blast and impact loads. In the future, the combination of polyurea—as a necessary protective material—and structural design is the trend for the joint development of materials and structures. For the research and development of polyurea, limited by the characteristics of polymer materials themselves, the improvement of polyurea with respect to the mechanical properties of strength and high temperature resistance are compounded with a variety of materials. This is also the current direction of high-performance materials.

It can be concluded that, with the progressive application of polyurea in the field of blast and impact protection, the application of polyurea can substantially improve the safety of structures, both in the protection of existing structures and in the design of new structures. With an emphasis on structural safety and the increasing threat of small-scale terrorism, the application of polyurea in blast and impact protection will gradually grow in the future. In view of the above, the application and research of polyurea in the field of blast and impact protection are bound to become a hot topic and to bring significant influence to the field of protection.

## Figures and Tables

**Table 1:** Mechanical properties of different brands of polyurea.

SL. No.	Brand	Year	Mechnical Property Details				Type	Ref.
			Tensile Strength (MPa)	Elongation (%)	Tear Strength (kN/m)	Shore Hardness		
1	EP JS	2008	20.34	350	87.5	-	aromatic	[38]
2	Dragon Shield-BC	2014	11.75	50	-	-	-	[28,29,30]
3	HM-VK	2016	10	500	-	-	-	[31]
4	Link-XS350	2019	22.39	163	-	60 ± 1 HD	-	[32,33,34]
5	SWD562	2019	16.5	160	-	-	-	[39]
6	SPUA 306	2019	24	400	85	85–95 HA	-	[14]
7	SPUA 307	2019	25	45	81	65–75 HD	-	[14]
8	AMMT-53	2020	25	50	90	70 HD	-	[15,35,36]
9	AMMT-55	2020	35	300	115	65 HD	-	[15,35]
10	AP103	2021	16	450	-	-	-	[16]
11	-	2021	18	350	45	90–96 HA	-	[40]
12	Qtech T26	2022	25.4	451.88	75.5	-	-	[13]

## Data Availability

Not applicable.
